# An Evaluation of Overall Goodness-of-Fit Tests for the Rasch Model

**DOI:** 10.3389/fpsyg.2018.02710

**Published:** 2019-01-10

**Authors:** Rudolf Debelak

**Affiliations:** Department of Psychology, University of Zurich, Zurich, Switzerland

**Keywords:** item response theory, Rasch model, item fit, type I error, power

## Abstract

For assessing the fit of item response theory models, it has been suggested to apply overall goodness-of-fit tests as well as tests for individual items and item pairs. Although numerous goodness-of-fit tests have been proposed in the literature for the Rasch model, their relative power against several model violations has not been investigated so far. This study compares four of these tests, which are all available in R software: *T*_10_, *T*_11_, *M*_2_, and the LR test. Results on the Type I error rate and the sensitivity to violations of different assumptions of the Rasch model (unidimensionality, local independence on the level of item pairs, equal item discrimination, zero as a lower asymptote for the item characteristic curves, invariance of the item parameters) are reported. The results indicate that the *T*_11_ test is comparatively most powerful against violations of the assumption of parallel item characteristic curves, which includes the presence of unequal item discriminations and a non-zero lower asymptote. Against the remaining model violations, which can be summarized as local dependence, *M*_2_ is found to be most powerful. *T*_10_ and LR are found to be sensitive against violations of the assumption of parallel item characteristic curves, but are insensitive against local dependence.

## Introduction

The application of models of item response theory (IRT) in psychological assessments requires a good fit of the chosen model to the data (see, for instance, Maydeu-Olivares, 2013). This is particularly true for the Rasch model (Rasch, 1960), which makes strong assumptions on the underlying item response process, which include local independence and parallel item response curves (see also Fischer, 1995). These assumptions lead to numerous unique characteristics of this model. For instance, the sum score is a sufficient statistic for a respondent's ability, the item and person parameters are separable, and comparisons of subpopulations are independent from the items used for this comparison, which is also named specific objectivity (McDonald, 1999). Numerous approaches for testing the model fit have been proposed (for overviews, see e.g., Glas and Verhelst, 1995; Maydeu-Olivares and Montaño, 2013). Among the described methods, overall goodness-of-fit tests can be discerned from tests which assess the model fit of individual items, item pairs, or persons. This article focuses on tests of the first type.

Among the overall goodness-of-fit tests, tests based on first-order statistics, which are built upon the comparison of expected and observed scores for individual items, can be discerned from tests based on second-order statistics, which are built upon the comparison of expected and observed scores for item pairs (van den Wollenberg, 1982; Glas, 1988; Suárez-Falcón and Glas, 2003). Following Suárez-Falcón and Glas, tests based on first-order statistics aim to be sensitive against violations of the assumption of parallel item characteristic curves. Tests based on second-order statistics, on the other hand, are designed to detect violations of the local independence assumption.

Some authors recommended the use of omnibus tests, like the *M*_2_ statistic of Maydeu-Olivares and Joe (2005), which aim to be sensitive against all model violations of practical relevance. This suggestion was supported by some simulation studies (e.g., Maydeu-Olivares and Montaño, 2013). In the classification of first- and second-order statistics, *M*_2_ is a second-order statistic.

Another practical concern of many global fit statistics for the Rasch model is that they are usually based on asymptotic theory, and may lead to unreliable results in small datasets. To assess the global model fit for small datasets, Ponocny (2001) suggested a non-parametric approach. Since the asymptotic distribution of a test statistic under the Rasch model does not need to be known in this framework, several additional test statistics were defined under this framework, of which only some have been evaluated in systematic simulation studies (e.g., Ponocny, 2001; Chen and Small, 2005; Koller et al., 2015).

Under a practical perspective, the problem of assessing the fit to the Rasch model has at least two aspects. The first aspect concerns the question which model test should be selected to test against a specific alternative model. The second aspect concerns the question which model violations can and cannot be detected by a specific model test. Both aspects make it necessary to evaluate the available test statistics with regard to their relative power against several alternative models of interest. Only few studies have evaluated the Type I error rate and power of the non-parametric model tests for larger datasets (an example being the unpublished diploma thesis of Jordan, 2010), and no studies seem to be available which compare non-parametric model tests for the Rasch model with omnibus tests like the *M*_2_ statistic.

This study therefore adds to the literature by comparing four available parametric and non-parametric first- and second-order statistics for the Rasch model with regard to their Type I error and their power against several alternative models in a broad simulation study. The evaluated test statistics were selected based on three criteria: First, all tests are currently available in published software and can therefore be easily applied to empirical datasets. Second, all tests were found to have power against several alternative IRT models in previous studies. Third, the tests are designed as global tests of model fit to the Rasch model. Based on these criteria, the following four test statistics were selected for this study: The LR statistic of Andersen (1973), the *T*_10_ and *T*_11_ test statistics of Ponocny (2001), and the *M*_2_ statistic of Maydeu-Olivares and Joe (2005).

The rest of this paper is organized as follows: In the following section Four Statistics for Testing the Fit of the Rasch Model, the four statistics are described. Section Method describes a variety of simulation studies for evaluating the various approaches, whose results are reported in section Software Used in This Study. In section Results, the application of all tests to an empirical dataset is illustrated. In section Empirical Data Example, all results are discussed and suggestions for practical applications are given.

## Four Statistics for Testing the Fit of the Rasch Model

As is widely known, the Rasch model uses the following item response function for describing the probability of a positive response of respondent *j* to item *i*:

(1) $$\begin{array}{r} P\left(X_{ji}=1|\theta_{j},\beta_{i}\right)=\frac{e_{j}^{\theta }-\beta_{i}}{1+e^{\theta_{j}-\beta_{i}}} \end{array}$$

In the context of psychological and educational testing, the item parameter β_*i*_ can be interpreted as a difficulty parameter for item *i*, whereas the person parameter θ_*j*_ can be interpreted as an ability parameter for respondent *j*. The following subsections provide an overview of the tests which are compared in this study.

### The LR Test of Andersen

This test was proposed by Andersen (1973) and further evaluated in a number of simulation studies (e.g., Suárez-Falcón and Glas, 2003). It aims at evaluating the stability of the item parameters β_*i*_ over different groups of respondents by comparing two conditional likelihoods. In order to calculate the test statistic, the original sample of test respondents is partitioned in *G* score groups. For each of the score groups and the total sample, the conditional likelihood of the observed responses is calculated. Given these likelihoods, the LR statistic is given by Glas and Verhelst (1995, p. 87):

$$\begin{array}{l} LR=2\left(\overset{G}{\underset{c=1}{\sum }}lnL_{c}\left(\hat{\beta_{c}}\right)-lnL\left(\hat{\beta }\right)\right) \end{array}$$

In this equation, $L\left(\hat{\beta }\right)$ denotes the conditional likelihood in the total sample based on the conditional maximum likelihood estimations of the item parameters, whereas $L_{c}\left(\hat{\beta_{c}}\right)$ denotes the conditional likelihood of the responses of group *c* based on the CML estimations of the item parameters in this score group. Under the Rasch model, the LR statistic is asymptotically χ^*2*^-distributed, with degrees of freedom equal to the number of parameters estimated in all respondent groups minus the number of parameters estimated in the total sample. LR is a first-order statistic. A widely used global test for the Rasch model is obtained if two groups, which consist of respondents with a raw score above or below the median raw score, are used for calculating LR. It was found to be sensitive against violations of the assumption of parallel item characteristic curves, but insensitive against multidimensionality (e.g., van den Wollenberg, 1982).

### The *M*_2_ Test of Maydeu-Olivares and Joe

This test is based on the general idea of using limited information statistics for assessing the global model fit. In contrast to the LR statistic, it is based on marginal maximum likehood estimation procedures for the item parameters, which assume a normal distribution for the person parameters. Maydeu-Olivares and Joe (2005) proposed a family of test statistics which are based on the moments of the multivariate Bernoulli distribution. It consists of statistics of the type (Maydeu-Olivares and Montaño, 2013, p. 119):

$$\begin{array}{l} M_{r}=N{\left(p_{r}-\pi_{r}\left(\hat{\theta }\right)\right)}^{\prime }{Ĉ}_{r}\left(p_{r}-\pi_{r}\left(\hat{\theta }\right)\right)\\ C_{r}=\Xi_{r}^{-1}-\Xi_{r}^{-1}\Delta_{r}{\left(\Delta_{r}^{\prime }\Xi_{r}^{-1}\Delta_{r}\right)}^{-1}\Delta_{r}^{\prime }\Xi_{r}^{-1} \end{array}$$

In these equations, *N* denotes the sample size, $\pi_{r}\left(\hat{\theta }\right)$ denotes the vector of moments of the multivariate Bernoulli distribution up to order *r*, *p*_*r*_ denotes the vector of sample joint moments up to order *r*. ${\hat{C}}_{r}$ denotes the evaluation of *C*_*r*_ at $\hat{\theta }$, whereas Ξ is the asymptotic covariance matrix of $\sqrt{N}(p_{r}-\pi_{r}\left(\hat{\theta }\right))$. Finally, $\Delta_{r}=\frac{\partial \pi_{r}(\theta )}{\partial \theta }$, with θ′ denoting the transpose of θ.

The basic idea of these statistics is the comparison of the observed moments for the multivariate Bernoulli distribution with those expected under a specific IRT model. Large deviations between the observed and expected moments indicate a model violation. Under the assumption of model fit, *M*_*r*_ follows a χ^*2*^-distribution with *s*×*q* degrees of freedom, where $s=\overset{r}{\underset{i=1}{\sum }}\left(\frac{n}{i}\right)$, with n being the number of items, and *q* the number of estimated item parameters. Of this proposed family of statistics, *M*_2_ was recommended by Maydeu-Olivares and Montaño (2013) for testing IRT models, since it does not only use bivariate information, but also has an accurate asymptotic approximation in small samples. *M*_2_ is a second-order statistic.

This test was recently evaluated in several studies (Maydeu-Olivares and Joe, 2005; Ranger and Kuhn, 2012; Maydeu-Olivares and Montaño, 2013). Their results indicated that it has power against violations of various assumptions made in commonly used IRT models, like the unidimensionality assumption, the assumption of local independence, and misspecifications of the form of the item characteristic curves.

### The Non-parametric Tests of Ponocny

Ponocny (2001) proposed a framework of tests for assessing the fit of the Rasch model in small samples. Since these tests do not require the estimation of person or item parameters, they are non-parametric. Tests in this framework are based on comparing the value of a test statistic, which represents a model violation of interest, against its distribution in a bootstrap sample of data matrices with the same marginal sums (i.e., the row and column sums) as the original dataset. This comparison leads to the calculation of *p*-values. Small *p*-values typically indicate a violation of the Rasch model. Statistically, these tests were shown to be uniformly most powerful tests of the Rasch model against more general IRT models. The bootstrap samples necessary for this procedure can be generated using algorithms proposed by Ponocny (2001), Chen and Small (2005), or Verhelst (2008). An important aspect of the non-parametric tests is that they are not feasible for large datasets because the related calculations become computationally too demanding.

We now consider two statistics proposed by Ponocny (2001) for assessing the overall model fit of the Rasch model in this non-parametric approach. The first test statistic, *T*_10_, is designed as a global test statistic for subgroup-invariance. It is calculated as $T_{10}=\sum_{ij}\left|N_{ij}^{\left(h\right)}N_{ji}^{\left(l\right)}-N_{ij}^{\left(l\right)}N_{ji}^{\left(h\right)}\right|$, where $N_{ij}^{\left(h\right)}$ denotes the number of respondents giving a positive response to item *i*, but not to *j*, and obtaining a raw score which corresponds to at least the median of the observed raw score distribution. $N_{ij}^{\left(l\right)}$ corresponds to the number of respondents showing the same response behavior, but obtaining a raw score below the median. This statistic can be considered as a non-parametric counterpart to the LR test and thus as a first-order statistic. Both tests were compared by Koller et al. (2015) in the context of the detection of differential item functioning (DIF) in small datasets, where *T*_10_ outperformed LR.

The second test statistic, *T*_11_, is designed as a global test for the violation of local stochastic independence. This statistic is calculated in two steps: First, the average inter-item correlation ρ_*ij*_ between all items *i* and *j* is calculated using the generated bootstrap samples, leading to an estimation of its expected value under the Rasch model. It is interesting to note that this step does not require the calculation of Rasch model parameters. *T*_11_ aims at comparing the expected values with its observed inter-item correlation *r*_*ij*_ and is calculated in a second step as $T_{11}=\underset{ij}{\sum }|r_{ij}-\rho_{ij}|$. Since *T*_11_ is based on comparing the observed and expected inter-item correlations, it is a second-order statistic. This test was found to be sensitive against multiple alternative models in an unpublished study of Jordan (2010).

### Goals of This Study

This study aims at comparing the tests based on the aforementioned first- and second-order statistics with regard to their Type I error rate and their power against several model violations (unidimensionality, local independence on the item level, equal item discrimination, zero as a lower asymptote for the item response function and invariance of the item parameters). Type I error rates and power rates will be reported for different conditions of sample size and test length.

These evaluations will be based on a variety of simulation studies, which will be described in the next section. Furthermore, the results of the four model tests in an empirical dataset will be compared.

## Methods

A variety of simulations studies was conducted to compare the four global model tests. Among the simulated datasets, there were four levels of sample size (100, 200, 500, and 1,000) and three levels of test length (10, 30, and 50). These sizes of the simulated respondent samples and item sets were chosen to be comparable to those typically reported in psychological research.

In all simulations, values for all model parameters were drawn from specific distributions, with the item parameters being fixed over all iterations and the person parameters being redrawn for each iteration. After having drawn all model parameters, standard functions from the eRm (Mair et al., 2015) and mirt (Chalmers, 2012) software packages were used to generate data under the various data generating models.

To obtain stable results, 5,000 iterations were run under each condition. The following subsection provides an overview of the data generating models used in this study. It is stated with each alternative model which tests were expected to be sensitive against it.

### Models Used for Data Generation

In a first simulation study, the empirical Type I error of all goodness-of-fit tests was investigated. In this study, the Rasch model, whose item response function is given by Equation (1), was used as data generating model. In each simulated dataset, the person parameters θ_*j*_ and the item difficulty parameter β_*i*_ were drawn from a standard normal distribution. All tests were expected to hold their nominal alpha level.

The second simulation study simulated a specific violation of local independence between two items. This type of model violation can occur as a result of similar item content or learning effects, or if one item is a prerequisite of another. The underlying idea of this model violation is inspired by the theory of knowledge spaces (e.g., Albert and Lukas, 1999; Doignon and Falmagne, 1999). It is assumed that there is a partial order in the item set, which is based on the knowledge or the abilities that are necessary for solving the individual items. As a consequence, it is not possible to solve difficult items from this order, which require more advanced knowledge, without being able to solve easier items, which require more basic knowledge. To simulate this model violation, all data were first generated based on the Rasch model as in the first simulation study and then altered subsequently. In this model violation, item 1, which had an item difficulty parameter of −0.626 in the data generation, was seen as a prerequisite of the more difficult item 2 with a difficulty parameter of 0.184. If the person parameters are drawn from a standard normal distribution, item 2 is typically solved by about 46% of the respondents. To simulate local dependence between these items, the response patterns of all respondents who gave a positive response to item 2, but not item 1, were considered. For 90% (corresponding to a major model violation) or 80% of these respondents (corresponding to a minor model violation), the response to item 1 was set to be a positive one. Simulations indicated that, as a consequence of this change, only 1.3% of all respondents provided a positive response to item 2 but not item 1 under the major model violation, whereas this was the case for 2.6% under the minor model violation, leading to a partial order between these items in a majority of the sample. This type of model violation resembles another model violation named surface local dependence (Chen and Thissen, 1997; Edwards et al., 2018) that is based on identical response patterns between pairs of items. The data generating model in our simulation study resembles surface response dependence because easier items in the simulated partial order are set to be solved when the more difficult items have been solved too, what makes the response vectors of these item pairs more similar than it is expected under the Rasch model. We expected the second-order tests *M*_2_ and *T*_11_ to be sensitive against this model violation, but not LR and *T*_10_.

The third simulation study aimed at the simulation of multidimensional data, which is another violation of the local independence assumption. Multidimensionality is commonly found in empirical datasets, and many methods for its detection have been proposed (e.g., Reckase, 2009). In these simulations, two person parameters θ_*j1*_ and θ_*j2*_ were drawn from a bivariate standard normal distribution with a covariance of *r* for each respondent j. Again, the item difficulty parameters were drawn from a standard normal distribution. After drawing all parameters, Equation (1) was used to generate the response matrix, with each person parameter used to generate responses for one half of the item set. *r* was set to 0.3 or 0.7, depending on the simulated condition. These conditions were chosen to mirror a weak or medium relationship between two latent traits. Similar designs were used in the studies of Maydeu-Olivares and Montaño (2013) and Suárez-Falcón and Glas (2003). Since this condition concerns another violation of local independence, we again only expected the second-order tests *M*_2_ and *T*_11_ to be sensitive against this model violation, and not LR and *T*_10_.

The fourth simulation study aimed at testing the power of the various model tests against a mixed Rasch model (Rost, 1990; Rost and von Davier, 1995), in which the parameter invariance assumption of the Rasch model is violated. Again, this is a common model violation whose detection is of high practical relevance (Holland and Wainer, 1993; Magis et al., 2010). In this simulation study, data were generated similar to the first simulation study, which investigated the Type I error rate. However, the item difficulty parameters of the first 20 or 40% of the items were reduced by 0.8, depending on the simulated condition, for 40% of the simulated respondents. The resulting two classes of respondents were assumed to be latent. This model violates the local independence assumption, since the probability of a correct response depends on the class to which the respondent belongs. This simulation study was inspired by previous studies on DIF detection in IRT studies (e.g., DeMars and Jurich, 2015; Kopf et al., 2015). As in the previous two simulation studies, we expected a sensitivity of *M*_2_ and *T*_11_ against this model violation.

The remaining simulation studies addressed violations of the assumption of parallel item characteristic curves. In these studies, data were generated under IRT models which generalize the Rasch model and have been regularly applied in empirical analyses.

In a fifth simulation study, the model tests were further evaluated using datasets generated from the 2PL model (Birnbaum, 1968). This model violates the assumption of equal item discrimination of the Rasch model. The probability of a positive response was therefore given by:

$$P\left(X_{ji}=1|\theta_{j},\alpha_{i},\beta_{i}\right)=\frac{e^{\alpha_{i}\left(\theta_{j}-\beta_{i}\right)}}{1+e^{\alpha_{i}\left(\theta_{j}-\beta_{i}\right)}}$$

Both the person parameters θ_*j*_ and the item difficulty parameters β_*i*_ were drawn from a standard normal distribution. Depending on the simulated condition, the item discrimination parameters α_*i*_ were drawn from a log-normal distribution lnlnN(0,0.09) or lnlnN(0,0.25), corresponding to a weak or strong violation of this assumption. Again, similar designs were used in the studies of Suárez-Falcón and Glas (2003) and Maydeu-Olivares and Montaño (2013). We expected all tests to be sensitive against this model violation, with the most powerful possibly being LR and *T*_10_, which are designed to detect this model violation.

The sixth simulation study aimed at evaluating the sensitivity of the four model tests against the violation of the assumption that the item characteristic curves have 0 as a lower asymptote. In empirical datasets, this assumption can be violated if respondents are able to guess the correct response. In the resulting one-parametric logistic (1PL) model with a pseudo-guessing parameter, the probability of a correct response is given by:

(2) $$P(X_{ji}=1|\theta_{j},\beta_{i},\gamma_{i})=\gamma_{i}+(1-\gamma_{i})\frac{e^{\theta_{j}-\beta_{i}}}{1+e^{\theta_{j}-\beta_{i}}}$$

As in the other models used in this simulation study, the item difficulty parameters β_*i*_ and the person parameters were drawn from a standard normal distribution, whereas the pseudo-guessing parameters γ_*i*_ were set to 0.1 or 0.25, depending on the simulated condition. Again, these values correspond to a weak or strong violation of this assumption. These values were drawn to mimic a situation where respondents with low ability would randomly select one response among ten (γ_*i*_ = 0.1) or four (γ_*i*_ = 0.25) response alternatives. Again, we expected all tests to be sensitive against this model violation with the most powerful possibly being LR and *T*_10_.

## Software Used in This Study

The free open framework for statistical computing R (R Core Team, 2017) was used in version 3.4.2. The following R packages were used for the data analysis: In order to apply the LR test of Andersen (1973) and the nonparametric tests of Ponocny (2001), the eRm package of Mair et al. (2015), version 0.16-0, was used. The *M*_2_ test of Maydeu-Olivares and Joe (2005) was applied using the mirt package of Chalmers (2012), versions 1.27.1 and 1.29. In the simulation studies involving the *T*_10_ and *T*_11_ statistic, the calculation of the *p*-values for these tests was based on 500 bootstrap samples for each dataset, using the default settings of the eRm package. The simulation studies were carried out using the SimDesign software package, versions 1.9 and 1.11 (Chalmers, 2018).

Under all conditions, an EM algorithm was used to estimate the item parameters in the mirt package. In the estimation of the difficulty parameters of the Rasch model, this algorithm converged within 500 iteration cycles under all conditions, which is also the default setting.

## Results

The results of the simulation studies are presented in three separate subsections. The first section contains results pertaining to the preservation of the nominal Type I error rate of each test. In the remaining two sections, the results on the sensitivity against alternative IRT models are summarized.

We first present results concerning IRT models which violate the local independence assumption of the Rasch model. These models encompass the two-dimensional Rasch model, the model with surface local dependence and the mixed Rasch model. We then present results on IRT models which violate the assumption of parallel item characteristic curves. These models include the 2PL model and the 1PL model with a pseudo-guessing parameter.

The results on the power of the four tests are presented as figures, which illustrate the power under conditions with major violations of the Rasch model. Readers who are interested in detailed results for all conditions are pointed to the Appendix, where these results are presented as tables.

### The Type I Error Rate

In general, the nominal alpha level of 0.05 was preserved under almost all conditions. For the tests based on LR and *T*_10_, the Type I error rate was between 0.04 and 0.06 under all conditions. For *T*_11_, the Type I error rate was between 0.056 and 0.063 for conditions with 30 items and between 0.067 and 0.070 for conditions with 50 items. For conditions with 10 items, the Type I error rate was between 0.048 and 0.052. There was no obvious relationship between an increase of the Type I error rate and the underlying sample size. For the test based on *M*_2_, an increased Type I error rate was generally observed for conditions with long tests and small samples. For this test, a Type I error rate above 0.060 was observed for conditions with samples of 100 or 200 respondents working on tests of length 30 or 50. Under all other conditions, the Type I error rate of this test was between 0.047 and 0.058. Analogous results were found for a nominal alpha level of 0.01. Detailed results are presented in Table A1 in the Appendix.

### Sensitivity Against Violations of Local Independence

**Figure 1:**
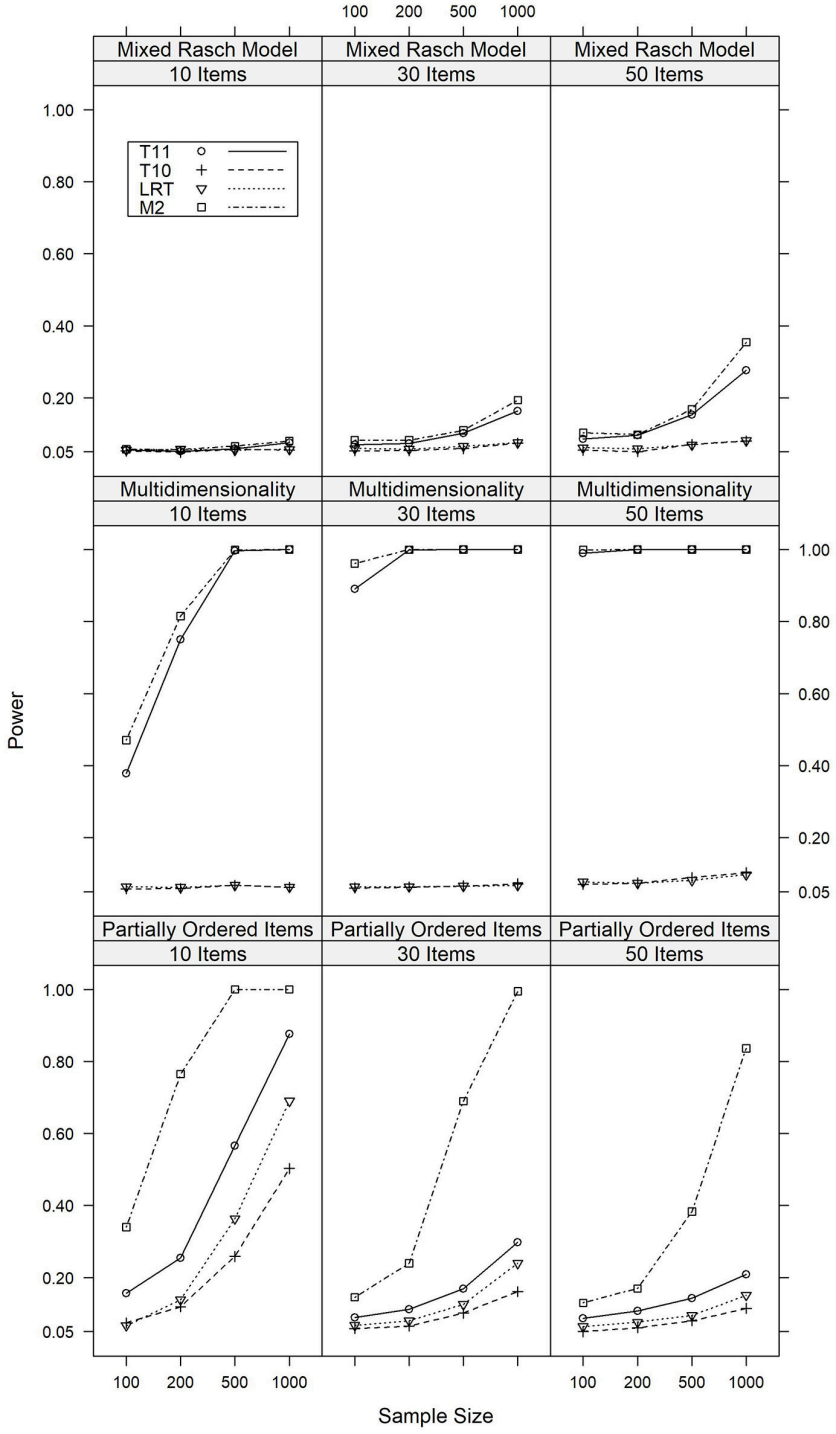
Power of the *T*_10_, *T*_11_, *M*_2_, and LR tests against major violations of local independence under various conditions of test length and sample size.

The power of the four tests against alternative models which violated the local independence assumption of the Rasch model are summarized in Figure 1 for major model violations, whereas detailed results are reported in Tables A2, A4 in the Appendix.

We will first discuss the results of the simulation study where the data were generated based on a multidimensional Rasch model with a correlation of 0.3 between the latent traits, indicating a major model violation. Under these conditions, *M*_2_ showed power rates between 0.471 and 1.000 and was overall most powerful. *T*_11_ was slightly less powerful under these conditions, with power rates between 0.378 and 1.000. For LR and *T*_10_, the power rates did not exceed 0.103. For a minor violation of multidimensionality (*r* = 0.7), analogous results were found. Detailed results are presented in Table A2.

Similar results were found for the model in which solving item 1 was a prerequisite for solving item 2 in a large part of the sample. This model thus simulated a partial order between these items. In case of a major model violation, the rate of significant results was between 0.130 and 1.000 for *M*_2_. The power of *T*_11_ was slightly smaller, with corresponding rates between 0.087 and 0.876. For LR and *T*_10_, these rates ranged between 0.050 and 0.690. For all tests, these rates were higher in datasets with a larger sample of respondents and smaller item sets. Similar results regarding the relative power of the tests were found in conditions with a minor model violation. In summary, *M*_2_ was the most powerful test against this model violation, although the other tests still had considerable power, particularly in datasets with small item sets. Detailed results are presented in Table A3.

*M*_2_ and *T*_11_ also had some power against differential item functioning, i.e., the mixed Rasch model, although the power rates were smaller than in the other simulation studies that investigated violations of local independence. If 40% of the items were affects by DIF for 40% of the respondents, the power rates ranged between 0.056 and 0.354 for *M*_2_ and between 0.051 and 0.277 for *T*_11_. For LR and *T*_10_, the power rates were 0.081 or below and therefore only slightly above the Type I error rate. Again, analogous results were found for a minor model violation. Detailed results are presented in Table A4.

In summary, in line with the expectations, the tests based on *T*_11_ and *M*_2_ were most powerful against violations of local independence, with *M*_2_ being slightly more powerful. LR and *T*_10_ had some power against local dependence on the level of item pairs and were insensitive against the mixed Rasch model and multidimensionality. This last finding is consistent with results reported by van den Wollenberg (1982), who used this finding to motivate the development of second-order statistics. The results for LR and *T*_10_ were not unexpected, since these statistics do not aim at detecting violations of the local independence assumption. It was overall surprising that none of the tests had much power against DIF, which is a model violation of high practical relevance.

### Sensitivity Against Violations of Parallel Item Characteristic Curves

**Figure 2:**
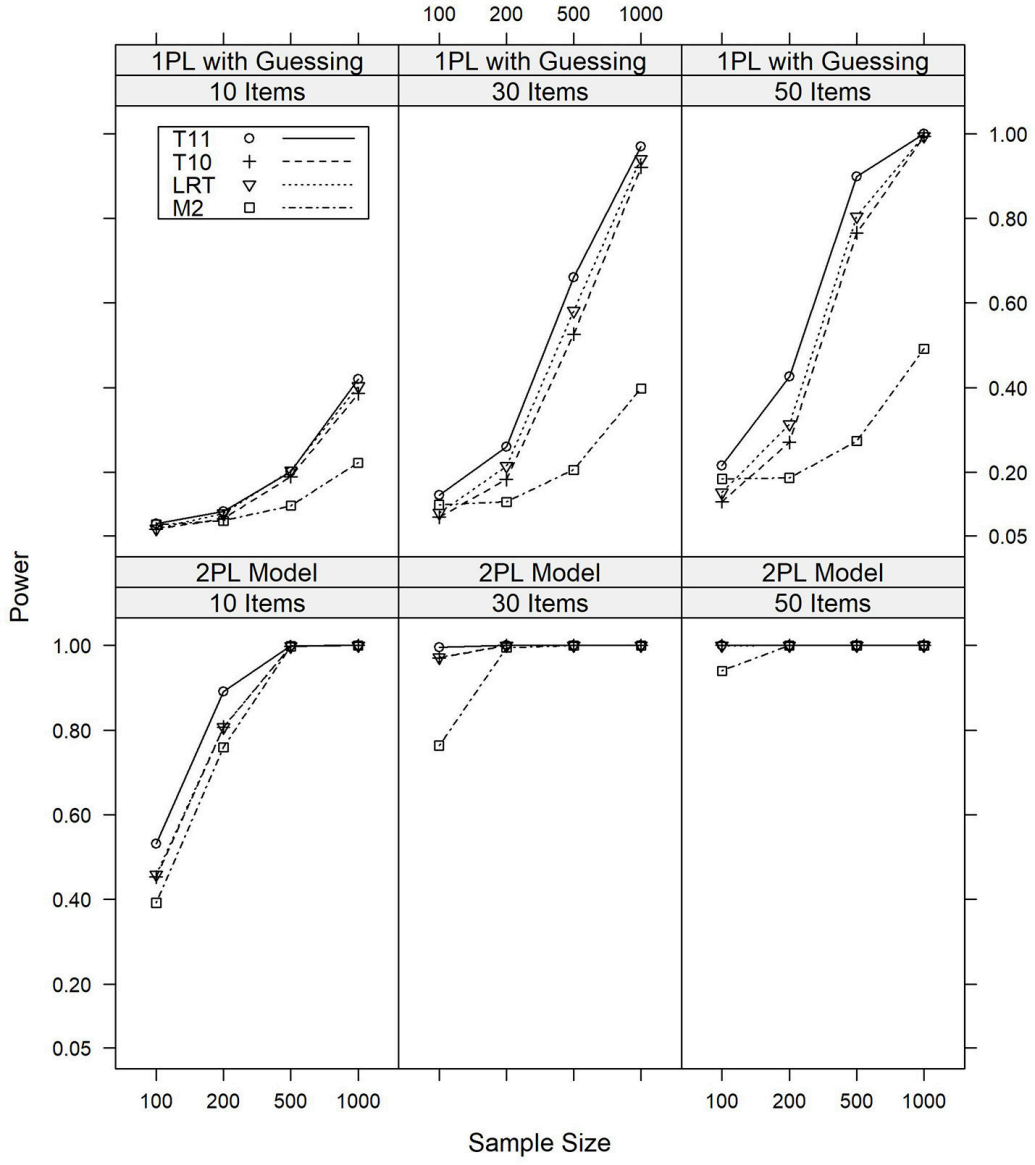
Power of the *T*_10_, *T*_11_, *M*_2_, and LR tests against major violations of the assumption of parallel item response curves under various conditions of test length and sample size.

In datasets generated from models which violated the assumptions of parallel item characteristic curves, a quite different pattern for the relative power of the four model tests was observed. The results for the major model violations are illustrated graphically in Figure 2, whereas detailed numerical results are given in Tables A5, A6 in the Appendix.

We first discuss the results on the power against the 2PL model. In summary, all tests were sensitive against the 2PL model, with many power rates being identical to or close to 1.000. Overall, *T*_11_ was the most powerful test against this model violation, with power rates of 0.532 or above for a major model violation and 0.194 or above for a minor model violation. LR and *T*_10_, which aim at detecting this model violation, are of comparable power and slightly less sensitive than *T*_11_, with power rates of 0.453 or above for major model violations and 0.176 or above for minor model violations. *M*_2_ was overall least powerful against this model violation, with power rates of 0.392 or more for major model violations and 0.147 or more for minor model violations. Detailed results are presented in Table A5.

Similar results were found for the 1PL model with a pseudo-guessing parameter. Again, *T*_11_ was overall most powerful, with LR and *T*_10_ being slightly less sensitive and *M*_2_ being least powerful. For major model violations, the power rates of *T*_11_ ranged between 0.079 and 0.999, depending on the sample size and test length. For LR and *T*_10_, the power rates were between 0.066 and 0.993. For *M*_2_, the power rates were between 0.076 and 0.492. Again, similar results were found for minor model violations. Table A6 presents detailed results.

In summary, *T*_11_ was most powerful against these model violations, which is a surprising result given that it is not designed to detect model violations of this type. *M*_2_, on the other hand, which is designed as an omnibus test, was unexpectedly found to be rather insensitive against a 1PL model with a pseudo-guessing parameter. In agreement with their aim, LR and *T*_10_ had considerable power to detect these model violations.

## Empirical Data Example

To further compare the results of the four tests, all methods were applied to an empirical dataset. This dataset was part of a larger dataset used by Janssen and Geiser (2010) to investigate solution strategies in mental rotation tasks, and was later made available in the TAM package for R (Kiefer et al., 2016). It contains the responses of 346 German students to eight items of a cube comparison task; a more detailed description of the sample and the test was provided by Janssen and Geiser. Other authors have found a good fit of similar tasks to the Rasch model (e.g., Tanzer et al., 1995). Janssen and Geiser found evidence for different solution strategies in these tasks, which may result in a violation of the assumption of local independence in the Rasch model, similar to the mixed Rasch model. Based on this hypothesis and the results of our simulation studies, we may select the *M*_2_ or *T*_11_ statistic to test the Rasch model against models with local dependence.

For both the *M*_2_ (*M*_2_ = 72.642, df = 27) and the *T*_11_ test, *p*-values smaller than 0.001 were obtained. In summary, these results indicate a violation of local independence, which is consistent with the hypothesis of Janssen and Geiser (2010). For the sake of completeness, we also report *p*-values for the other two tests: A *p*-value of 0.029 was found for the LR test (LR = 15.633, df = 7), and for the *T*_10_ test, a *p*-value of 0.016 was calculated.

## Discussion

An important question in the practical application of any statistical test is whether the empirical Type I error equals the nominal Type I error rate. It was found that the nominal level of significance was preserved by the LR and the *T*_10_ tests, whereas the Type I error rate for *T*_11_ was increased for longer tests and for *M*_2_ in datasets with small samples working on long tests. Given these results, we will now discuss whether each test is sensitive against the model violations it was designed to detect, and whether it is sensitive against other model violations.

Although the *T*_11_ test was designed as a global test for local independence, it was found to be also sensitive against alternative models which violate the assumption of parallel item characteristic curves. These results indicate that it should rather be seen as a test that is sensitive against various alternative models. Although *T*_11_ was designed for small samples, the reported results indicate that this test has a slightly increased Type I error rate in longer tests, which had not been previously reported in the literature.

The results for *M*_2_ indicate that this test is particularly useful to detect local dependence when testing the Rasch model. Compared to the other three tests, it has overall less power against violations of the assumption of parallel item characteristic curves, which was not reported by previous studies. Another important difference between *M*_2_ and the other tests is that *M*_2_ is based on MML estimation and thus assumes a normal distribution of the person parameters.

The LR and *T*_10_ tests were found to be particularly sensitive against alternative models which violate the assumption of parallel item characteristic curves. Although these first-order statistics do not aim to be sensitive against a violation of local independence on the level of item pairs, the results indicate that these tests also have some power against this model violation in small tests. The results also show that these tests are not sensitive against multidimensionality or the mixed Rasch model.

As was already stated in the introduction, the problem of selecting an appropriate global fit test for the Rasch model involves two related, but distinct questions. The first question concerns the selection of a fit statistic to test against a specific alternative model. Generally, not all model violations detected by overall goodness-of-fitness tests need to be of practical relevance (cf. van der Linden and Hambleton, 1997, p. 16), but the application of insensitive model tests might result in overlooking model violations of practical relevance. A sensible strategy for avoiding this pitfall might entail the formulation of alternative IRT models which correspond to practically significant model violations, the selection of model tests which are most powerful against these models, and an estimation of the necessary sample size for testing against these models with sufficient power. The evaluation of the relative power of the available model tests against various alternative models, as it was done in this study, is a necessary step in the development of tools for power analysis and sample size planning in the field of IRT. We also note that a number of approaches have been proposed in the literature to assess whether a model misfit has practical significance, for instance, using model residuals (Sinharay and Haberman, 2014) or graphical model checks (Sinharay, 2005).

The second question concerns the interpretation of the results of the model tests. Our results indicate that not all test statistics are specifically sensitive against the model violations they were designed to detect (for instance *T*_11_, which is not only sensitive against violations of local independence), and that no model test has power against all model violations considered here. For practical test evaluations, this leads to the recommendation to carry out several model tests if there are multiple plausible alternative models. These tests can be complemented by fit statistics for individual persons, items or item pairs to assess the fit of a given dataset to the Rasch model. A similar advice was given by van der Linden and Hambleton (1997, p. 16) for assessing the fit of the 2PL and 3PL models, and by Maydeu-Olivares and Liu (2015). Glas and Verhelst (1995, chap. 5.2.3) give an overview of tests on the level of individual items, whereas Kim et al. (2011) evaluate several general tests of model fit on the level of item pairs, which can also be applied in the context of the Rasch model. Methods for the detection of DIF effects in the context of the Rasch model entail tests based on focal and reference groups (for an overview: Magis et al., 2010), mixed Rasch models (Rost, 1990; Rost and von Davier, 1995) and Rasch trees (Strobl et al., 2015). The application of these tests may help in detecting the exact nature of the model violation.

Another question of practical relevance, which was not addressed in this study, concerns the problem of assessing model fit in the presence of missing data (for an overview: Mislevy, 2017). Of the four tests considered in this study, the statistic of the LR test can be calculated even in the presence of missing data. On the other hand, this is not directly possible for *M*_2_, *T*_11_, and *T*_10_. The mirt software package uses data imputation (e.g., Schafer and Graham, 2002) to allow the calculation of *M*_2_ even in the presence of missing data. However, missing data might be generated by various different processes (Mislevy, 2017), and the use of data imputation and similar methods may or may not lead to a bias in the test statistics, depending on the specific process. This important topic is left to future research.

## Data Availability Statement

The data sets generated for this study as well as the data generating code can be obtained upon request from the author.

## Author Contributions

The author confirms being the sole contributor of this work and has approved it for publication.

### Conflict of Interest Statement

The author declares that the research was conducted in the absence of any commercial or financial relationships that could be construed as a potential conflict of interest.
