# Supplementary material for: An Evaluation of Overall Goodness-of-Fit Tests for the Rasch Model
Source: Front Psychol. 2019 Jan 10;9:2710. doi: 10.3389/fpsyg.2018.02710 (PMC6335387; doi:10.3389/fpsyg.2018.02710)
Supplement: Supplementary file 1 [file Data_Sheet_1.pdf]

An Evaluation of Overall Goodness-of-Fit Tests for the Rasch Model – Online Appendix

Rudolf Debelak

This appendix presents the detailed results of the simulation studies that were presented in the main text. It therefore allows readers to check the Type I error rate or power of the Rasch model tests under all simulated conditions. Table A1 presents the results on the Type I rate, Table A2 presents the power against multidimensionality, Table A3 addresses the power against local independence in one item pair, and Table A4 presents the power against the mixed Rasch model. Table A5 summarizes the power against the 2PL model and Table A6 against the 1PL model with a pseudo-guessing parameter.

Table A1. The empirical Type I error rate of the model tests in datasets with i items and N persons for two nominal alpha levels.

	i	N	LR	M_2	T_{10}	T_{11}
α = 0.01	10	100	0.011	0.010	0.010	0.009
		200	0.012	0.012	0.010	0.011
		500	0.009	0.011	0.007	0.009
		1000	0.009	0.012	0.008	0.011
	30	100	0.012	0.015	0.010	0.011
		200	0.012	0.013	0.012	0.013
		500	0.014	0.011	0.011	0.009
		1000	0.010	0.012	0.010	0.014
	50	100	0.013	0.022	0.013	0.013
		200	0.010	0.015	0.007	0.016
		500	0.010	0.009	0.010	0.017
		1000	0.010	0.008	0.009	0.012
α = 0.05	10	100	0.052	0.053	0.051	0.052
		200	0.051	0.056	0.047	0.051
		500	0.050	0.047	0.047	0.048
		1000	0.053	0.051	0.050	0.052
	30	100	0.050	0.071	0.050	0.061
		200	0.056	0.061	0.052	0.063
		500	0.049	0.058	0.049	0.056
		1000	0.049	0.055	0.051	0.063
	50	100	0.056	0.083	0.052	0.068
		200	0.055	0.069	0.048	0.068
		500	0.049	0.055	0.047	0.070
		1000	0.049	0.049	0.049	0.067

Table A2. The power of the model tests against multidimensionality for various values of r in datasets with i items and N persons. Values larger than 0.5 are written in bold.

	i	N	LR	M_2	T_{10}	T_{11}
r = 0.3	10	100	0.064	0.471	0.058	0.378
		200	0.062	**0.815**	0.059	**0.750**
		500	0.068	**0.999**	0.068	**0.997**
		1000	0.063	**1.000**	0.062	**1.000**
	30	100	0.063	**0.961**	0.061	**0.890**
		200	0.064	**1.000**	0.063	**0.999**
		500	0.066	**1.000**	0.066	**1.000**
		1000	0.067	**1.000**	0.072	**1.000**
	50	100	0.077	**0.999**	0.071	**0.990**
		200	0.073	**1.000**	0.074	**1.000**
		500	0.082	**1.000**	0.089	**1.000**
		1000	0.097	**1.000**	0.103	**1.000**
r = 0.7	10	100	0.052	0.115	0.045	0.091
		200	0.055	0.199	0.051	0.142
		500	0.045	0.484	0.045	0.364
		1000	0.055	**0.855**	0.053	**0.751**
	30	100	0.057	0.345	0.056	0.195
		200	0.059	**0.632**	0.054	0.410
		500	0.053	**0.991**	0.051	**0.931**
		1000	0.052	**1.000**	0.054	**1.000**
	50	100	0.058	**0.597**	0.052	0.341
		200	0.058	**0.918**	0.054	**0.683**
		500	0.053	**1.000**	0.057	**0.997**
		1000	0.057	**1.000**	0.058	**1.000**

Table A3. The power of the model tests against local dependence in one item pair in datasets with i items and N persons. Values larger than 0.5 are written in bold.

	i	N	LR	M_2	T_{10}	T_{11}
Partial item order in 98.6% of sample	10	100	0.067	0.340	0.074	0.156
		200	0.138	**0.765**	0.119	0.254
		500	0.363	**1.000**	0.259	**0.566**
		1000	**0.690**	**1.000**	**0.503**	**0.876**
	30	100	0.067	0.145	0.059	0.089
		200	0.080	0.239	0.066	0.112
		500	0.126	**0.690**	0.101	0.169
		1000	0.240	**0.995**	0.161	0.298
	50	100	0.064	0.130	0.050	0.087
		200	0.076	0.170	0.060	0.108
		500	0.095	0.383	0.080	0.143
		1000	0.151	**0.836**	0.115	0.209
Partial item order in 97.4% of sample	10	100	0.073	0.253	0.077	0.139
		200	0.114	**0.563**	0.095	0.204
		500	0.265	**0.991**	0.208	0.441
		1000	**0.503**	**1.000**	0.390	**0.742**
	30	100	0.061	0.127	0.059	0.078
		200	0.075	0.180	0.071	0.100
		500	0.104	**0.504**	0.086	0.141
		1000	0.159	**0.933**	0.119	0.226
	50	100	0.064	0.117	0.056	0.082
		200	0.066	0.131	0.057	0.097
		500	0.086	0.273	0.071	0.130
		1000	0.115	**0.643**	0.095	0.169

Table A4. The power of the model tests against the mixed Rasch model in datasets with i items and N persons.

	i	N	LR	M_2	T_{10}	T_{11}
40% DIF items	10	100	0.055	0.057	0.052	0.055
		200	0.057	0.056	0.049	0.051
		500	0.055	0.066	0.057	0.059
		1000	0.057	0.081	0.055	0.076
	30	100	0.059	0.083	0.053	0.069
		200	0.057	0.083	0.053	0.074
		500	0.066	0.110	0.059	0.102
		1000	0.076	0.193	0.074	0.164
	50	100	0.061	0.103	0.055	0.086
		200	0.059	0.098	0.050	0.096
		500	0.069	0.168	0.070	0.153
		1000	0.081	0.354	0.080	0.277
20% DIF items	10	100	0.050	0.058	0.051	0.052
		200	0.056	0.052	0.052	0.050
		500	0.046	0.049	0.045	0.048
		1000	0.048	0.060	0.047	0.054
	30	100	0.051	0.069	0.052	0.058
		200	0.053	0.069	0.051	0.067
		500	0.055	0.064	0.053	0.068
		1000	0.047	0.080	0.046	0.075
	50	100	0.063	0.094	0.053	0.074
		200	0.056	0.075	0.054	0.074
		500	0.063	0.094	0.058	0.091
		1000	0.050	0.133	0.049	0.111

Table A5. The power of the model tests for the Rasch model against the 2PL model for different distributions of α_i in datasets with i items and N persons. Values larger than 0.5 are written in bold.

	i	N	LR	M_2	T_{10}	T_{11}
$\alpha_i \sim ln\mathcal{N}(0,0.25)$	10	100	0.459	0.392	0.453	**0.532**
		200	**0.807**	**0.760**	**0.807**	**0.891**
		500	**0.998**	**0.998**	**0.998**	**1.000**
		1000	**1.000**	**1.000**	**1.000**	**1.000**
	30	100	**0.971**	**0.764**	**0.970**	**0.995**
		200	**1.000**	**0.995**	**1.000**	**1.000**
		500	**1.000**	**1.000**	**1.000**	**1.000**
		1000	**1.000**	**1.000**	**1.000**	**1.000**
	50	100	**1.000**	**0.940**	**1.000**	**1.000**
		200	**1.000**	**1.000**	**1.000**	**1.000**
		500	**1.000**	**1.000**	**1.000**	**1.000**
		1000	**1.000**	**1.000**	**1.000**	**1.000**
$\alpha_i \sim ln\mathcal{N}(0,0.09)$	10	100	0.181	0.147	0.176	0.194
		200	0.390	0.298	0.347	0.419
		500	**0.854**	**0.779**	**0.807**	**0.894**
		1000	**0.996**	**0.993**	**0.987**	**0.998**
	30	100	**0.460**	0.203	0.430	**0.569**
		200	**0.868**	0.423	**0.825**	**0.933**
		500	**1.000**	**0.957**	**0.999**	**1.000**
		1000	**1.000**	**1.000**	**1.000**	**1.000**
	50	100	**0.827**	0.357	**0.794**	**0.949**
		200	**0.996**	**0.713**	**0.993**	**1.000**
		500	**1.000**	**1.000**	**1.000**	**1.000**
		1000	**1.000**	**1.000**	**1.000**	**1.000**

Table A6. The power of the model tests against a 1PL model with a pseudo-guessing parameter for different values of γ_i in datasets with i items and N persons. Values larger than 0.5 are written in bold.

	i	N	LR	M_2	T_{10}	T_{11}
$\gamma_i = 0.25$	10	100	0.067	0.076	0.066	0.079
		200	0.103	0.085	0.091	0.108
		500	0.204	0.121	0.190	0.201
		1000	0.403	0.223	0.386	0.420
	30	100	0.104	0.123	0.094	0.146
		200	0.215	0.130	0.183	0.260
		500	**0.581**	0.206	**0.527**	**0.661**
		1000	**0.940**	0.398	**0.920**	**0.969**
	50	100	0.152	0.185	0.131	0.216
		200	0.314	0.187	0.271	0.427
		500	**0.804**	0.274	**0.766**	**0.899**
		1000	**0.994**	0.492	**0.993**	**0.999**
$\gamma_i = 0.10$	10	100	0.065	0.057	0.059	0.063
		200	0.076	0.068	0.068	0.076
		500	0.103	0.076	0.099	0.107
		1000	0.183	0.101	0.174	0.184
	30	100	0.072	0.081	0.068	0.089
		200	0.106	0.066	0.095	0.134
		500	0.227	0.095	0.202	0.295
		1000	0.450	0.125	0.416	**0.604**
	50	100	0.083	0.094	0.073	0.121
		200	0.129	0.080	0.115	0.186
		500	0.314	0.086	0.282	0.465
		1000	**0.640**	0.125	**0.607**	**0.838**
